# A Pipeline for Discovering Inhibitors of Wildtype Tau Seeded Aggregation

**DOI:** 10.1002/alz70855_104344

**Published:** 2025-12-24

**Authors:** Matthew Reid, Anna Brown, William A McEwan

**Affiliations:** ^1^ University of Cambridge, Cambridge, Cambridgeshire, United Kingdom; ^2^ UK Dementia Research Institute at University of Cambridge, Cambridge, United Kingdom; ^3^ University of Cambridge, Cambridge, United Kingdom

## Abstract

**Background:**

Tauopathies, including Alzheimer's disease (AD), are defined by the pathological aggregation of tau proteins, a process linked to neurodegeneration. Misfolded tau spreads in a prion‐like manner, recruiting native tau and propagating across brain regions. Existing models often rely on mutant tau or recombinant seeds, which fail to fully mimic tau conformations seen in AD, limiting therapeutic discovery.

**Methods:**

We developed a screening pipeline using HEK cells expressing wildtype 3R/4R tau seeded with AD post‐mortem tissue‐derived aggregates, ensuring disease relevance. As a complementary approach, primary mouse neurons expressing mutant tau were seeded with recombinant aggregates, offering a comparative model for validating hits. Additionally, an iPSC‐derived neuronal model of seeded wildtype tau aggregation is under development to further enhance physiological relevance.

**Results:**

The pipeline successfully identified inhibitors of tau aggregation. In HEK cells, these inhibitors blocked AD‐derived tau seeding, while in the primary neuron model, they prevented recombinant tau‐induced aggregation. This dual approach demonstrates the inhibitors’ efficacy across wildtype and mutant tau systems, bridging model relevance and translational potential.

**Conclusion:**

This study establishes a robust pipeline for tau aggregation inhibitor discovery, integrating wildtype tau relevance with mutant tau models. The addition of an iPSC‐neuronal model will further enhance the physiological fidelity of the pipeline. The mechanisms of the successful compounds are currently being determined to understand their mode of action, and will progress toward lead optimisation and preclinical evaluation, advancing therapeutic development for AD and related tauopathies.

